# The effects of AG1® supplementation on the gut microbiome of healthy adults: a randomized, double-blind, placebo-controlled clinical trial

**DOI:** 10.1080/15502783.2024.2409682

**Published:** 2024-10-01

**Authors:** Michael B. La Monica, Betsy Raub, Shelley Hartshorn, Ashley L. Gustat, Jodi Grdic, Trevor O. Kirby, Jeremy R. Townsend, Jen Sandrock, Tim N. Ziegenfuss

**Affiliations:** a The Center for Applied Health Sciences, Canfield, OH, USA; b AG1, Research, Nutrition, and Innovation, Carson City, NV, USA; c Concordia University Chicago, Health & Human Performance, River Forest, IL, USA

**Keywords:** Gut health, microbiome, gastrointestinal symptoms, dietary supplementation, probiotics, prebiotics

## Abstract

**Background:**

This study aimed to examine the effect of a commercially available multi-ingredient powder (AG1^Ⓡ^) on the gut microbiome and assess the impact of AG1^Ⓡ^ on GI tolerability and other clinical safety markers in healthy men and women.

**Methods:**

Using a double-blind, randomized, two-arm, placebo-controlled, parallel design, we examined a 4-week daily supplementation regimen of AG1^Ⓡ^ vs. placebo (PL). Fifteen men and 15 women provided stool samples for microbiome analysis, questionnaires for digestive quality of life (DQLQ), and completed visual analog scales (VAS) and Bristol stool charts to assess stool consistency and bowel frequency before and after the 4-week intervention. Participant’s blood work (CBC, CMP, and lipid panel) was also assessed before and after the 4-week intervention. Alpha diversity was determined by Shannon and Chao1 index scores and evaluated by a two-way ANOVA, beta diversity in taxonomic abundances and functional pathways was visualized using partial least squares-discriminant analyses and statistically evaluated by PERMANOVA. To identify key biomarkers, specific feature differences in taxonomic relative abundance and normalized functional pathway counts were analyzed by linear discriminant analysis (LDA) effect size (LEfSe). Questionnaires, clinical safety markers, and hemodynamics were evaluated by mixed factorial ANOVAs with repeated measures. This study was registered on clinicaltrials.gov (NCT06181214).

**Results:**

AG1^Ⓡ^ supplementation enriched two probiotic taxa (*Lactobacillus acidophilus* and *Bifidobacterium bifidum*) that likely stem from the probiotics species that exist in the product, as well as *L.* *lactis* CH_LC01 and *Acetatifactor* sp900066565 ASM1486575v1 while reducing *Clostridium* sp000435835. Regarding community function, AG1^Ⓡ^ showed an enrichment of two functional pathways while diminishing none. Alternatively, the PL enriched six, but diminished five functional pathways. Neither treatment negatively impacted the digestive quality of life via DQLQ, bowel frequency via VAS, or stool consistency via VAS and Bristol. However, there may have been a greater improvement in the DQLQ score (+62.5%, *p* = 0.058, d = 0.73) after four weeks of AG1^Ⓡ^ supplementation compared to a reduction (−50%) in PL. Furthermore, AG1^Ⓡ^ did not significantly alter clinical safety markers following supplementation providing evidence for its safety profile.

**Conclusions:**

AG1^Ⓡ^ can be consumed safely by healthy adults over four weeks with a potential beneficial impact in their digestive symptom quality of life.

## 1. Introduction

A growing body of literature details the impact of gut health on overall health. Many diseases have been linked to changes in the gut microbial profile, often due to an imbalance between beneficial and harmful bacteria, as well as decreased microbial diversity [1,2]. Indeed, the gut microbiota is pivotal in the digestion and absorption of nutrients, which helps maintain host health, particularly regulating reactions for immune function, bioenergetics, and growth [3]. There is a bidirectional interaction between the gut and micronutrients. On one hand, microbes utilize micronutrients for growth and support biological function, thus dietary supplementation with specific vitamins (i.e. Vitamin B, C, D, E) and minerals (i.e. calcium, iron, zinc, magnesium, phosphorus) may shape the gut microbiome [3,4]. On the other hand, the intestinal microbial makeup can control micronutrient absorption and synthesize vitamins [3,4].

Given the profound impact the gut microbiome elicits on human health, there is growing interest in strategies to improve gut health including the consumption of prebiotics, probiotics, or synbiotics. Prebiotics are non-digestible food components that can support short-chain fatty acid (SCFA) production. SCFAs are metabolites resulting from the fermentation of dietary fiber and protein fermentation in the gut that play a significant role in gut health by promoting cell migration, protecting against intestinal infection [5]. Additionally, they have been shown to improve intestinal integrity and homeostasis by regulating the immune system and reducing inflammation [5]. Probiotics are live microorganisms that can confer a health benefit on the host when administered in adequate amounts [6]. Similarly, probiotics can beneficially modulate the gut through the expansion of bacteria and yeast, thereby increasing the mucosal layer to improve gut barrier function keeping pathogens out and stimulating immune cells [7]. Synbiotics are defined as the combination of prebiotics and probiotics that amplify and promote health benefits to the host.

AG1^Ⓡ^ is a novel synbiotic dietary supplement blend that includes vitamins, minerals, prebiotics, probiotics, and phytonutrients. Currently, it is reported that 1 in 20 people in the US report using non-food pre, pro, or synbiotic products [8]. The probiotic species in AG1^Ⓡ^ are some of the most well-studied, coming from the genera *Lactobacillus* and *Bifidobacterium*. Specifically, bifidobacteria are abundant in the human gut microbiota and aid in protection against pathogens [9], modulate the host immune response to promote an anti-inflammatory environment [10], produce vitamins and SCFAs, and enhance the intestinal gut barrier [11,12]. Likewise, *L. acidophilus* has been shown to reduce the duration of diarrhea and improve stool consistency [13]. Additionally, some polyphenols may act as prebiotics, providing a potential for these phytochemicals to impact the growth of beneficial bacteria, the reduction of unwanted bacteria, and the promotion of greater microbial diversity [14–16]. Preclinical data has shown that AG1^Ⓡ^ exerts a prebiotic effect by increasing beneficial metabolites resulting from fermentation (i.e. total SCFA production), enriches beneficial bacteria (i.e. *F. prausnitzii*, *Microcoleaceae*, *Waltera intestinalis*, and *Arthrospira*) and lead to beneficial shifts in the polar fecal metabolome (e.g. metabolites in the serotonin metabolism pathway) in a Simulator of the Human intestinal Microbial Ecosystem^Ⓡ^ (SHIME) model [17–19]. These modifications in the microbiome likely provided protection to a Caco-2 model of the intestinal barrier after an inflammatory challenge [20]. Although these data were collected using an *ex vivo* model, the SHIME model offers several advantages. It aims to accurately replicate the components of the human GI tract, including sourcing human stool samples to generate a human microbiome *ex vivo.*

According to cross-sectional data from 2017–2018, over 57% of US adults reported using a dietary supplement in the past month [21]. The most common types are multivitamin and mineral (MVM) supplements, with 24% of adults aged 20–39, 29.8% aged 40–59, and 39.4% aged 60 or older reporting taking MVM supplements in the past month [21]. However, some consumers express concern regarding potential GI side effects associated with MVM or dietary supplement consumption including diarrhea, nausea, abdominal cramps, and other GI disturbances [22–24]. GI symptoms already burden nearly two out of three Americans [25], and these symptoms can interfere with their quality of daily life. AG1^Ⓡ^ has been shown to be more easily digestible than a multivitamin tablet (i.e. high solubility), resulting in good bioaccessibility and bioavailability in an *in vitro* model of the digestive tract [26]. In this GI model, AG1^Ⓡ^ also demonstrated low levels of gas production compared to other studies investigating high gas-producing foods [17].

While these preclinical data are promising, *in vitro* models have inherent limitations when translating to *in vivo* effects in humans given the myriad of factors that can influence GI physiology. Thus, the primary aim of this study was to examine the effect of AG1^Ⓡ^ on the gut microbiome of healthy men and women. Additionally, this study sought to assess the effect of AG1^Ⓡ^ on GI tolerability and other clinical safety markers as secondary and tertiary aims, respectively. We hypothesized that AG1^Ⓡ^ would beneficially alter fecal microbiome structure (i.e. enrichment of beneficial changes in key taxa, and functional metagenomics), be well tolerated, and produce no negative alterations in clinical safety markers.

## 2. Materials and methods

### 2.1. Experimental design

**Figure 1.**
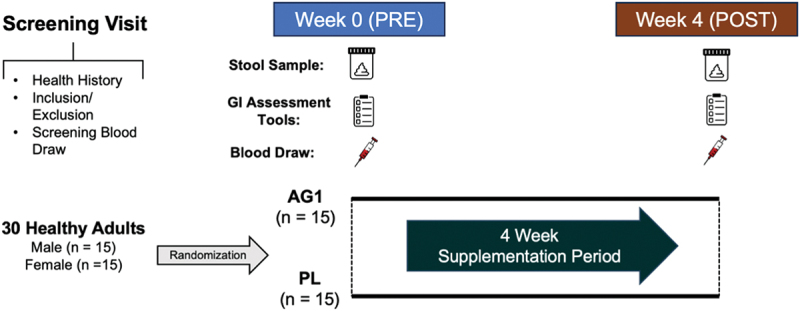
Study design overview. GI: Gastrointestinal; PL: Placebo.

This was a double-blind, randomized, two-arm, placebo-controlled clinical trial in which participants visited the laboratory on three occasions (one screening visit, one baseline visit, and a four-week visit). This study was conducted according to the guidelines in the Declaration of Helsinki of 1975 and all procedures involving human subjects were approved by the WCG IRB on 6/22/23 (#AG-01-0623). Written informed consent was obtained from all subjects prior to enrollment and was registered on clinicaltrials.gov (NCT06181214). During the initial screening visit, each participant’s medical history and blood work (CBC, CMP, and lipid panel) were assessed, and baseline diet was evaluated. During visits two (Week 0-pre) and three (Week 4-post) subjects completed subjective questionnaires including visual analog scales (VAS) that assessed stool consistency and frequency of bowel movements, digestion-associated quality of life questionnaire (DQLQ), Bristol stool chart to subjectively assess stool consistency, and the Framingham physical activity questionnaire. On visit three (after 4 weeks) participants also underwent follow-up blood work (CBC, CMP, lipid panel). After baseline testing, participants were given their respective supplement [i.e. placebo (PL) or AG1^Ⓡ^] to take daily for four weeks. Two stool collection kits were also provided at visit 2 (Week 0-pre). Participants were instructed to collect a stool sample at home before starting their daily supplement regimen and again within three days of their visit three (Week 4-post) (see Figure 1).

### 2.2. Participants

30 healthy adults (15 men and 15 women) completed all study visits (See Table 1 for subject characteristics). Participants were eligible for this study if they were in good health as determined by physical examination and medical history, between the ages of 18 and 50 years, and had a body mass index (BMI) of 18.5–29.9 kg•m^−2^. Prior to participation, all participants indicated their willingness to comply with all aspects of the experimental and supplement protocol. Participants were excluded if they had: (a) a history of diabetes or endocrine disorder; (b) a history of malignancy in the previous 5 years except for non-melanoma skin cancer (basal cell cancer or squamous cell cancer of the skin); (c) prior GI bypass surgery; (d) known GI or metabolic diseases that might impact nutrient absorption or metabolism (e.g. short bowel syndrome, diarrheal illnesses, history of colon resection, gastro paresis, Inborn Errors of Metabolism); (e) any chronic inflammatory condition or disease; (f) a known allergy to any of the ingredients in the supplement or the placebo; (g) or were currently participating in another research study with an investigational product or have been in another research study in the past 30 days; (h) or had a previous medical diagnosis of asthma, gout, or fibromyalgia; (i) were current smokers or smoking within the past month; (j) alcohol consumption (more than 2 standard alcoholic drinks per day or more than 10 drinks per week) or drug abuse or dependence within the past 6 months; (k) history of hyperparathyroidism or an untreated thyroid condition; (l) were pregnant women, women trying to become pregnant, women less than 120 days postpartum or nursing women; had any other diseases or conditions that, in the opinion of the medical staff, could confound the primary endpoint or place the participant at increased risk of harm if they were to participate; (m) did not demonstrate a verbal understanding of the informed consent document.

**Table 1.** Participant characteristics.

	AG1 (*N* = 15)	PL (*N* = 15)
Age (years)	39.2 ± 8.6	38.1 ± 9.2
Men (#)	7	8
Women (#)	8	7
Height (cm)	171.4 ± 7.7	169.2 ± 12.9
Weight (kg)	74.4 ± 11.9	76.8 ± 14.7
Body Mass Index (kg/m^2^)	25.2 ± 2.9	26.6 ± 2.4
Systolic Blood Pressure (mm Hg)	114.0 ± 14.2	116.1 ± 11.1
Diastolic Blood Pressure (mm Hg)	73.5 ± 11.4	74.2 ± 7.0
Resting Heart Rate (bpm)	65.5 ± 8.5	64.5 ± 9.1

Participants were instructed to follow their normal diet and activity patterns throughout their participation in the study and were required to complete a 24-hour diet record prior to arriving at the laboratory for their initial screening visit. Participants were given a copy of this dietary record and instructed to duplicate all food and fluid intake 24 hours prior to each subsequent laboratory visit. In addition, participants were asked to complete 3-day diet logs prior to starting their supplementation regimen and again during the last week of their supplementation regimen to document participants’ diets before and after the intervention. Prior to each subsequent visit, participants were asked to verbally confirm their 24-hour prior diet adherence. In addition to replicating food and fluid intake for 24 hours prior, study participants were also asked to refrain from exercise, alcohol, and caffeine 24 hours prior, and arrive 10 hours fasted to all testing sessions. Again, these instructions were all verbally confirmed at the beginning of each study visit.

### 2.3. Questionnaires

100-mm anchored VAS were completed before (pre) and after (post) the four-week supplement regimen. VAS were anchored with “Loose stool,” or “Less frequent than normal” and “Hard stool,” “or “More frequent than normal” and assessed subjective ratings of stool consistency and frequency of my bowl movements. The validity and reliability of VAS to assess fatigue and energy have been previously established [27] and reported [28,29].

The DQLQ is a 9-item questionnaire that assesses how often digestive symptoms interfere with quality of life over the past seven days. Specifically, it captures associations between digestive symptoms and physical activity, dietary intake, worries or concerns, emotional well-being, social interaction, and physical appearance [30]. Possible scores range from 0 to 9 with a higher score indicating worse digestion associated with quality of life. The DQLQ was specifically designed for healthy populations who experience GI symptoms (such as the sample in the current study) and it has been previously shown to be valid and reliable [30]. The DQLQ was assessed before and after the 4-week intervention.

The Bristol stool chart is a 7-option (score 1–7) scale form that allows participants to identify how loose or hard their stool consistency is and serves as a predictor of intestinal transit time [31]. It is represented with pictures and accompanying descriptors that suggest constipation with a low score or diarrhea with a high score. Meanwhile scores of 3–4 are considered normal stools. Type I correlates to separate hard lumps, like nuts, Type II correlates to sausage-shaped but lump, Type III correlates to like a sausage or snake but with cracks on its surface, Type IV correlates to like a sausage or snake, smooth and soft, Type V correlates to soft blobs with clear-cut edges, Type VI correlates to fluffy pieces with ragged edges, a mushy stool, and Type VII correlates to watery, no solid pieces [31]. Practically this chart can be used in patients/subjects with GI complications to evaluate the response to a treatment. The Bristol stool chart was assessed before and after the 4-week intervention.

The Framingham physical activity questionnaire assesses the average hours of participation in sleep, rest, occupational and extracurricular activities over a typical 24-hour period [32]. It was used to ensure participants maintained their habitual physical activity habits throughout the study. A higher value is indicative of more physical activity.

### 2.4. Supplement protocol

All supplements were prepared and packaged in coded generic single sachets for consumption throughout the study protocol. These white sachets arrived at the lab with a printed code to blind the researchers and to distinguish between treatments and lot numbers. Participants orally ingested a PL (12 g maltodextrin + flavoring) or a AG1^Ⓡ^ (AG1; Athletic Greens International, Carson City, NV, USA) daily for four weeks. The nutritional information and ingredients for AG1^Ⓡ^ are available online at https://drinkag1.com (accessed on 13 December 2023) and the full product has undergone evaluation and verification via NSF testing (Ann Arbor, MI, USA) to ensure the product meets strict quality, purity, safety, and label accuracy standards [33]. Using a parallel design, half of the subjects were randomly assigned to receive PL while the other half were assigned to receive AG1 matched for sex. Subject enrollment order was used to randomize the subjects in order to get an equal number of men and women within each group. A random number generator was used (https://www.calculatorsoup.com/calculators/statistics/random-number-generator.php) to set the enrollment order randomization. Participants were given a supplement sign off sheet to record their daily adherence in an easily viewable 4 week check off diagram and instructed to return their empty sachets to display proof of compliance and adherence (most subjects were > 96% compliant with two > 89% compliant).

### 2.5. Anthropometric and other resting measures

Standing height was determined using a wall-mounted stadiometer and body weight was measured using a Seca 767^TM^ Medical Scale (at each visit). Resting heart rate and blood pressure were measured using an automated blood pressure cuff (Omron HEM-780) during visits two (baseline) and three (week 4).

### 2.6. Stool collection kits

Two at-home stool kits (DNA/RNA Shield Fecal Collection Kit, Zymo Research) were given to each participant at visit two (pre). Each stool kit supplied a collection tube with an integrated spoon and DNA stabilizer, a clear sample bag, and a bubbled sealed envelope along with detailed instructions for collection. Participants were instructed to collect fecal samples before the intervention and near the end of their 4-week intervention (i.e. within three days before visit three). Stool kits were returned to the laboratory for mailing to a third-party laboratory for microbiome analysis (Zymo Research, Irvine, CA, USA). Participants were asked to collect their two samples at approximately the same time of day.

### 2.7. Microbiome analyses

Genomic DNA was extracted from baseline and week four stool samples using the ZymoBIOMICS^Ⓡ^ 96 MagBead Kit (Zymo Research, Irvine, CA). Genomic DNA libraries were prepped using the Illumina^Ⓡ^ DNA Library Prep Kit (Illumina, San Diego, CA) with up to 500 ng DNA input and used unique 10 base pair (bp) dual-index barcodes with NextEra^Ⓡ^ adapters. To sequence the libraries, either NovaSeq X or 6000^Ⓡ^ kits were used (Illumina, San Diego, CA). All kits were used adhering to the manufacturer’s instructions. Controls were used to ensure there were no issues with the kits used. Positive controls used were the ZymoBIOMICS^Ⓡ^ Microbial Community Standard (Zymo Research, Irvine, CA) and ZymoBIOMICS^Ⓡ^ Microbial Community DNA Standard (Zymo Research, Irvine, CA) for the DNA extraction and library prep phases, respectively. Blank reactions were used as negative controls.

Once raw sequences were obtained, trimming was applied using Trimmomatic-0.33 [34]. The trimming process applied a quality trim with a 6 bp window size and a quality cutoff of 20, and reads with a size lower than 70 bp were removed. The Centrifuge microbial classification engine [35] was used to profile the microbial composition using known bacterial, viral, fungal, and human genome datasets. Abundance data at the strain-level was extracted from the centrifuge outputs for further bioinformatic analysis. Functional pathways were identified and annotated using the MetaCyc database [36]. Taxonomic abundances were normalized to relative abundance and functional pathway counts were normalized to counts per million units to account for comparisons between samples with different sequencing depths.

### 2.8. Adverse events (AEs)

All adverse events (all local and systemic non-serious and serious) were monitored by the researchers and evaluated and assessed through reports coded using the Medical Dictionary for Regulatory Activities (MedDRA). In the event of an AE, its intensity would have been graded according to the protocol-defined criteria based on the Common Terminology Criteria for Adverse Events (CTCAE) Version 5.0, 2017.

### 2.9. Statistical analyses

Statistical analyses pertaining to the microbiome were conducted in R 4.2.2 (https://www.r-project.org/) [37]. Alpha diversity (Shannon Diversity Index and Chao1 Diversity Index scores) [38] were determined using the fossil package [39] and statistically evaluated by two-way ANOVA. Beta diversity (Heterogeneity) in taxonomic abundances and functional pathways were visualized using partial least squares-discriminant analyses (PLS-DA) using the mixOmics package (http://mixomics.org/) [40,41] and statistically evaluated by adonis, a PERMANOVA approach based on McArdle and Anderson, 2001 [42], using the vegan package (https://cran.r-project.org/web/packages/vegan/index.html) [43]. To identify key biomarkers, specific feature differences in taxonomic relative abundance and normalized functional pathway counts were analyzed by linear discriminant analysis (LDA) effect size (LEfSe) [44]. Only features with an LDA score greater than 1.5 and associated p-values less than 0.05 were reported [45,46].

Data on questionnaires, clinical safety markers, and hemodynamics are presented as means ± standard deviation and the primary statistical approach employed was mixed factorial ANOVA with repeated measures on time to assess group (AG1 vs. PL), time (week 0 and week 4), and group x time interaction effects. Sidak post hoc comparisons were made upon statistical significance in the mixed factorial ANOVA model. All variables were tested for normality using results from a Shapiro-Wilk test. Changes from baseline (deltas) were calculated and independent t-tests were computed to evaluate between-group changes using 95% confidence intervals, p-values, and effect sizes for the primary outcome measures. Effects sizes (Cohen’s d) were also used to determine the magnitude of change on all significant post hoc outcomes with a small effect size being ≥ 0.2, a medium effect being ≥ 0.5, and a large effect being ≥ 0.8 [47]. A significance level of 0.05 was used for all statistical determinations (as an industry standard), while p-values between 0.051 and 0.10 were deemed a trend. Given the observed effect size in this study based on the DQLQ we achieved a power of 97%. These statistical analyses were conducted using GraphPad Prism version 10.1.1.

## 3. Results

15 women and 15 men completed all study visits with at least 89% compliance to the intervention Table 1.

### 3.1.1. Microbiome analysis

#### 3.1.1.1. Community structure

**Figure 2.**
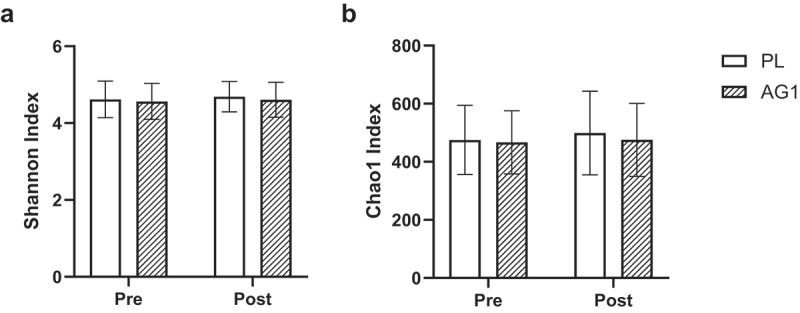
Alpha diversity analyses on fecal microbiota before and after supple-mentation. (a) Shannon diversity index values; (b) Chao1 diversity index values.

Changes in species richness and/or evenness (alpha diversity) were examined using the Shannon and Chao1 diversity indices (Figure 2). There was no significant interaction between time and treatment for the Shannon (*p* = 0.589) or Chao1 (*p* = 0.547) indices. Shannon Diversity Index did yield a significant effect of time (*p* = 0.03), but not for treatment (*p* = 0.686). Chao1 Diversity Index did not yield a significant effect of time (*p* = 0.21) or treatment (*p* = 0.721).

**Figure 3.**
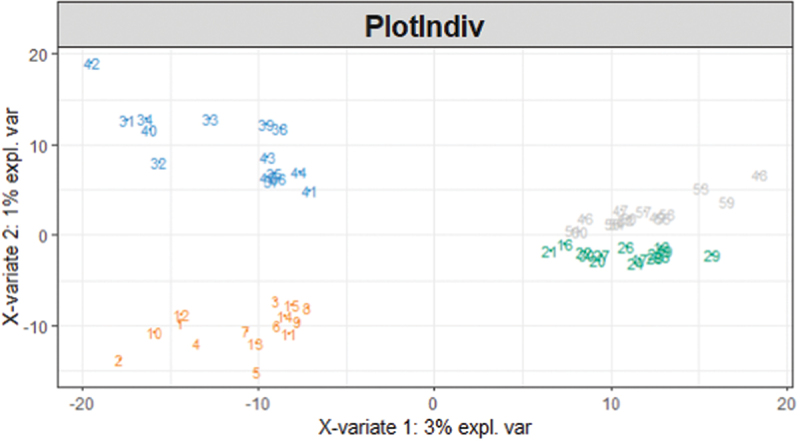
A PLS-DA ordination visualizes the effects of treatment and time influenced on community structural heterogeneity. Numbers 1 through 15 (orange) refer to the AG1® treated individuals at baseline sampling and numbers 16 through 30 (green) refer to the placebo (PL) treated individuals at baseline. Numbers 31 through 45 (blue) refer to the AG1® treated individuals after the experimental period, and 46 through 60 refer to PL-treated individuals after the experimental period.

Heterogeneity in relative abundances (beta diversity) was visualized and demonstrated clear clustering centered around both treatment group and time point (Figure 3). Despite this, results from the PERMANOVA suggested no differences at baseline (*p* = 0.105, R^2^ = 0.03762) or at the end of the experimental period (*p* = 0.119, R^2^ = 0.03764) between the two treatment groups. Moreover, approximately 96% of the variation was explained by another variable. Subsequently, the effect of the donor was analyzed and suggested to explain approximately 91% of the variation. Due to clear, significant differences within the community structure of the treatments, the AG1 group and placebo group were analyzed independently of each other with respect to individual differences within taxa.

**Figure 4.**
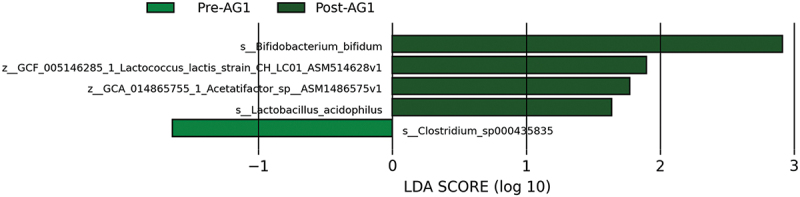
Results from the taxonomic LEfSe analysis for the AG1 treated group.

**Figure 5.**
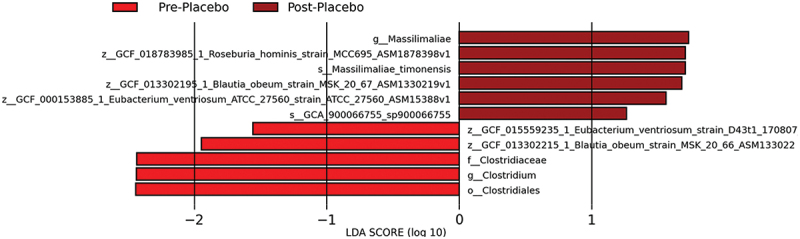
Results from the taxonomic LEfSe analysis for the placebo (PL) treated group.

LEfSe was used to identify key taxa acting as biomarkers for differences between the baseline samples and end of the experimental period samples. Within the AG1 group, four taxa were enriched while one taxon was diminished (Figure 4). Within the placebo-treated group, six taxa were enriched while five taxa were diminished (Figure 5).

#### 3.1.1.2. Community function

**Figure 6.**
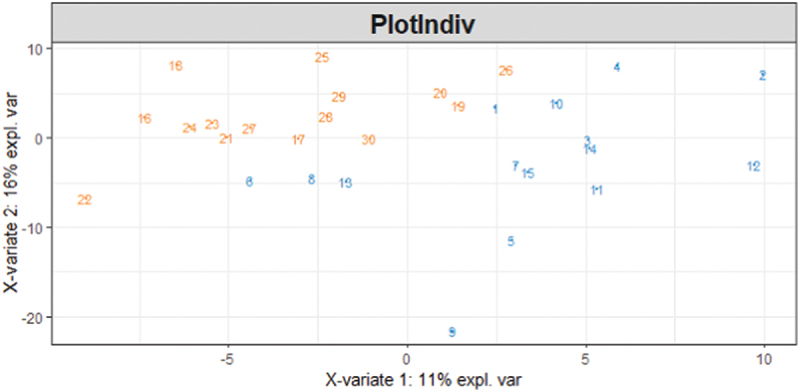
A PLS-DA ordination visualizes the effects of AG1® and placebo (PL) treated groups on community functional heterogeneity following four weeks of supplementation. Numbers 1 through 15 (orange) refer to the AG1® treated group. Numbers 16 through 30 (blue) refer to the PL-treated group.

At the end of the experimental period, heterogeneity in specific functional pathway counts was visualized (Figure 6). To assess changes in functional heterogeneity, PERMANOVA analysis was used to evaluate the treatment’s effect. No significant difference was observed in the predicted metabolic function of the gut microbiota between the two treatment groups (*p* = 0.434, R^2^ = 0.03285).

**Figure 7.**
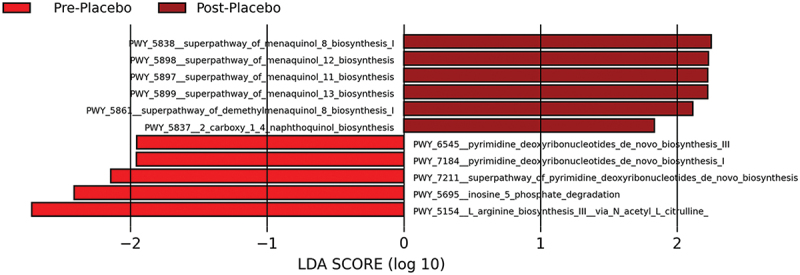
Results from the functional LEfSe analysis for the placebo (PL) treated group.

Based on the PLS-DA data demonstrating clear clustering between the timepoints for both treatment groups, LEfSe was used to identify key taxa acting as biomarkers for differences between the baseline samples and end of the experimental period samples. Within the AG1 group, two functional pathways were enriched [i.e. palmitate biosynthesis type I fatty acid synthase (MetaCyc, PWY_5994) and guanosine ribonucleotides de novo biosynthesis (MetaCyc, PWY_7221)] while no pathways were diminished. Within the PL group, six pathways were enriched while five pathways were diminished (Figure 7).

### 3.1.2. Questionnaires

**Figure 8.**
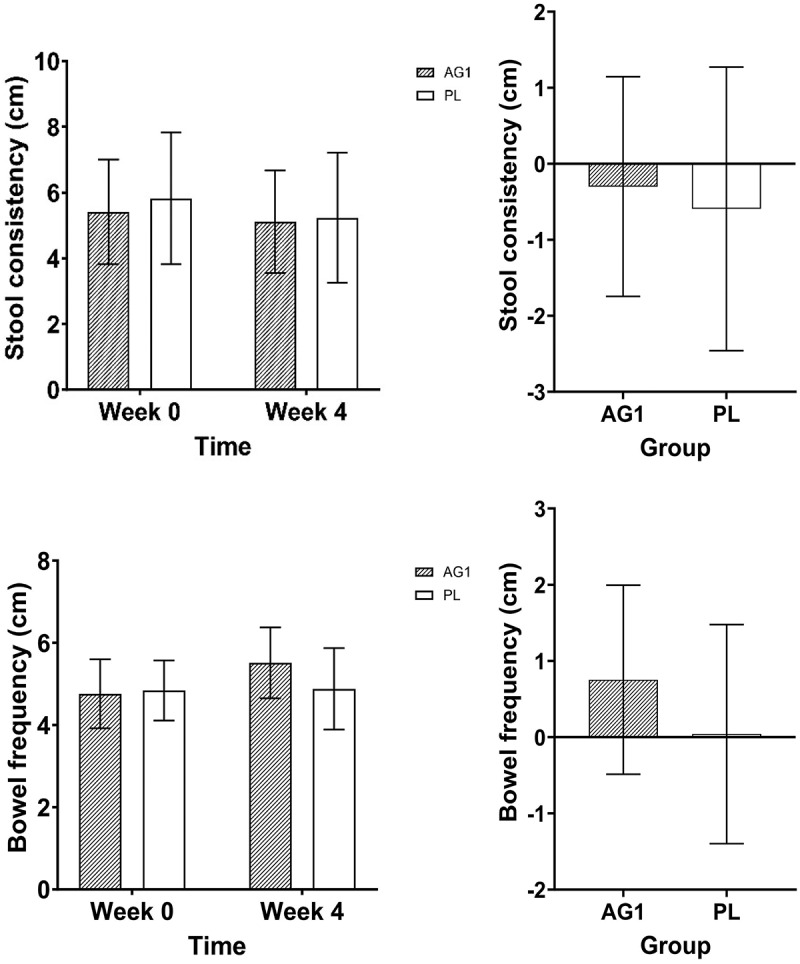
Raw data for stool consistency and bowel frequency from VAS, and their respective deltas before and after supplementation. Data are shown as Mean + SD.

There were no differences between groups over time for stool consistency (Time: *p* = 0.154, Group: *p* = 0.650, Group x Time: *p* = 0.634) or bowel frequency (Time: *p* = 0.117, Group: *p* = 0.171, Group x Time: *p* = 0.157). In addition, there were no differences between groups in the change score for stool consistency (*p* = 0.634) or bowel frequency (*p* = 0.157) (see Figure 8).

**Figure 9.**
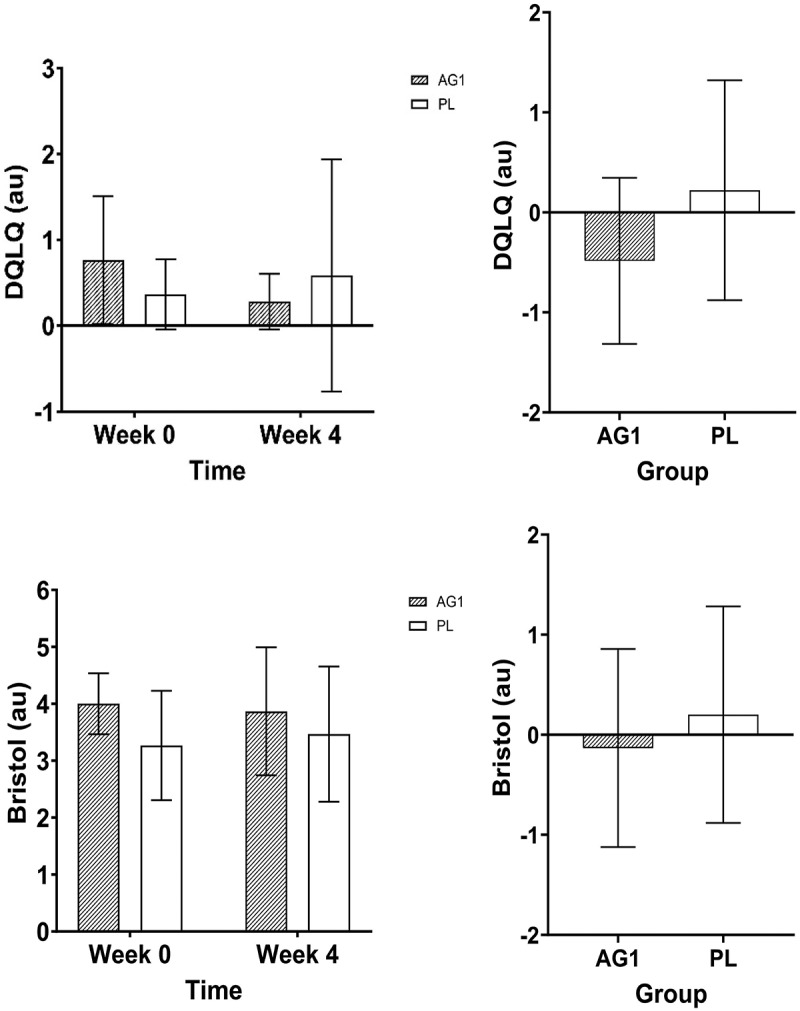
Raw data for the Digestion-associated Quality of Life Questionnaire (DQLQ) and Bristol stool scale and their respective deltas before and after supplementation. Data are shown as Mean + SD.

There was a group by time interaction trend (*p* = 0.057) for the DQLQ score, however there were no significant post hoc outcomes. However, there was a trend for differences between groups for the change score in DQLQ (mean difference = 0.71 au, 95%CI: −0.025 to 1.44, *p* = 0.058, d = 0.73). It appeared that AG1^Ⓡ^ reduced (i.e. improved) DQLQ score by 62.5% while PL had increased (i.e. reduced) DQLQ score by 50% from baseline to week 4. (see Figure 9).

There was a group by time interaction trend (*p* = 0.075) for the Bristol stool score. Post hoc analysis showed that AG1 group may have had a greater score (~21.2%) than PL at baseline (mean difference = 0.73 au, 95%CI: −0.09 to 1.56, *p* = 0.090, d = 0.53). Alternatively, there were no differences between groups for the change score in Bristol stool (*p* = 0.386) (see Figure 9).

There were no differences between groups over time for the Framingham (Time: *p* = 0.383, Group: *p* = 0.383, Group x Time: *p* = 0.793) showing that both groups maintained their usual physical activity habits throughout the study.

### 3.1.3. Clinical safety markers, hemodynamics, diet, body weight, and adverse events

There was a significant interaction for Creatinine (*p* = 0.013). Post hoc analysis showed that Creatinine increased from baseline to week four by ~ 6.7% in the PL group (mean difference = −0.053 mg/dL, 95%CI: −0.097 to −0.008, *p* = 0.020, d = 0.72). There was a significant interaction for eGFR (*p* = 0.013). Post hoc analysis showed that eGFR decreased from baseline to week four by ~ 5.6% in the PL group (mean difference = 5.6 mL/min/1.73, 95%CI: 0.27 to 10.9, *p* = 0.038, d = 0.64). There was an interaction trend for Bilirubin being elevated in the placebo group (*p* = 0.086), however there were no significant post hoc outcomes (Table 2).

**Table 2.** Complete metabolic panel and complete blood count.

Variable	Group	PRE (Week 0)	POST (Week 4)	Main Effect for Time	Main Effect for Group	Group x Time
Complete Metabolic Panel						
Glucose (mg/dL)	AG1	89.9 ± 5.2	88.9 ± 7.1	0.425	0.483	0.803
	PL	91.3 ± 7.7	90.7 ± 7.9			
BUN (mg/dL)	AG1	15.1 ± 4.1	15.1 ± 4.9	0.897	0.436	0.796
	PL	14.1 ± 3.9	13.9 ± 3.7			
Creatinine (mg/dL)	AG1	0.96 ± 0.20	0.94 ± 0.20	0.217	0.643	0.013*
	PL	0.89 ± 0.18	0.95 ± 0.19^a^			
BUN/Creatinine ratio (au)	AG1	15.8 ± 4.9	16.3 ± 5.4	0.875	0.761	0.323
	PL	15.9 ± 3.7	15.2 ± 5.0			
eGFR (mL/min/1.73)	AG1	90.9 ± 13.6	93.2 ± 12.6	0.315	0.233	0.019*
	PL	100.3 ± 11.7	94.7 ± 14.4^a^			
Sodium (mmol/L)	AG1	139.6 ± 1.5	139.3 ± 1.4	0.334	0.529	>0.999
	PL	139.9 ± 2.3	139.6 ± 1.5			
Potassium (mmol/L)	AG1	4.42 ± 0.28	4.36 ± 0.24	0.389	0.222	0.916
	PL	4.32 ± 0.24	4.28 ± 0.24			
Chloride (mmol/L)	AG1	103.1 ± 1.7	102.1 ± 1.8	0.012*	0.584	0.377
	PL	103.1 ± 1.4	102.7 ± 1.7			
CO_2_ (mmol/L)	AG1	23.0 ± 2.1	23.4 ± 1.1	0.372	0.707	0.765
	PL	23.3 ± 2.0	23.5 ± 2.2			
Calcium (mg/dL)	AG1	9.38 ± 0.27	9.44 ± 0.26	0.618	0.068#	0.322
	PL	9.60 ± 0.26	9.58 ± 0.33			
Total Protein (g/dL)	AG1	7.0 ± 0.4	6.9 ± 0.4	0.869	0.738	0.784
	PL	7.0 ± 0.2	7.0 ± 0.3			
Albumin (g/dL)	AG1	4.55 ± 0.29	4.52 ± 0.25	0.342	0.058#	0.931
	PL	4.71 ± 0.20	4.67 ± 0.20			
Globulin (g/dL)	AG1	2.41 ± 0.25	2.41 ± 0.24	0.547	0.226	0.651
	PL	2.27 ± 0.29	2.31 ± 0.36			
A/G ratio (au)	AG1	1.90 ± 0.25	1.91 ± 0.22	0.565	0.063#	0.460
	PL	2.12 ± 0.33	2.07 ± 0.35			
Bilirubin (mg/dL)	AG1	0.63 ± 0.47	0.57 ± 0.46	>0.999	>0.999	0.086#
	PL	0.57 ± 0.25	0.63 ± 0.32			
Alkaline Phosphatase (IU/L)	AG1	60.1 ± 19.0	63.1 ± 22.4	0.068#	0.059#	0.334
	PL	76.4 ± 21.5	77.3 ± 22.5			
AST (IU/L)	AG1	22.7 ± 6.7	23.1 ± 7.3	0.825	0.943	0.875
	PL	22.7 ± 5.9	22.7 ± 7.5			
ALT (IU/L)	AG1	22.0 ± 11.0	23.1 ± 12.3	0.326	0.781	0.951
	PL	20.9 ± 10.4	21.9 ± 11.9			
Complete Blood Count						
WBC (x10E3/uL)	AG1	5.6 ± 1.7	5.7 ± 1.5	0.986	0.735	0.905
	PL	5.5 ± 1.1	5.5 ± 1.1			
RBC (x10E6/uL)	AG1	4.77 ± 0.53	4.75 ± 0.54	0.492	0.993	0.857
	PL	4.77 ± 0.52	4.74 ± 0.51			
Hemoglobin (g/dL)	AG1	14.2 ± 1.6	14.1 ± 1.5	0.542	0.388	0.793
	PL	14.6 ± 1.4	14.6 ± 1.4			
Hematocrit (%)	AG1	42.1 ± 3.9	41.4 ± 4.3	0.108	0.630	0.692
	PL	42.7 ± 3.9	42.3 ± 3.8			

*Indicates a statistically significant effect/interaction between treatments (*p* ≤ 0.05). ^#^ Indicates a trend for a main effect or interaction between treatments (*p* ≤ 0.10). ^a^ Indicates a statistically significant difference vs. Week 0 (*p* ≤ 0.050).

BUN: Blood Urea Nitrogen; eGFR: estimated Glomerular Filtration Rate; AST: Aspartate aminotransferase; ALT: Alanine transaminase; WBC: White Blood Count; RBC: Red Blood Count.

There was a significant main effect of time for Chloride (*p* = 0.012). There was a group trend for Calcium (*p* = 0.068), Albumin (*p* = 0.058), and A/G ratio (*p* = 0.063). There was a group (*p* = 0.059) and a time (*p* = 0.068) trend for Alkaline Phosphatase.

There were no differences between groups over time for WBC, RBC, hemoglobin, hematocrit, glucose, BUN, BUN:Creatinine ratio, sodium, potassium, CO_2_, total protein, globulin, AST, ALT, total cholesterol, triglycerides, HDL, VLDL, LDL, LDL/HDL, total/HDL, SBP, DBP, or body weight (*p* > 0.100). Table 3. There was a significant main effect of time for HR (*p* = 0.032). Table 3. There was a main effect of time for protein (*p* = 0.031), but no other group, time, or group x time differences for total calories, carbohydrates, or fats (*p* > 0.10).

**Table 3.** Cardiometabolic health markers and body weight.

Variable	Group	PRE (Week 0)	POST (Week 4)	Main Effect for Time	Main Effect for Group	Group x Time
Total Cholesterol (mg/dL)	AG1	187.2 ± 31.3	185.3 ± 22.6	0.853	0.459	0.471
	PL	194.0 ± 38.1	197.3 ± 46.4			
Triglycerides (mg/dL)	AG1	84.1 ± 39.1	83.1 ± 34.5	0.362	0.584	0.519
	PL	95.6 ± 45.2	87.5 ± 44.2			
HDL (mg/dL)	AG1	60.3 ± 18.3	61.1 ± 17.7	0.916	0.944	0.562
	PL	60.5 ± 19.4	59.9 ± 18.4			
VLDL (mg/dL)	AG1	15.9 ± 6.5	15.4 ± 5.7	0.324	0.618	0.647
	PL	17.5 ± 7.7	16.2 ± 7.6			
LDL (mg/dL)	AG1	111.1 ± 33.3	108.7 ± 27.0	0.611	0.482	0.181
	PL	116.0 ± 36.9	121.1 ± 38.2			
LDL/HDL (au)	AG1	2.1 ± 1.0	2.0 ± 0.8	0.597	0.655	0.344
	PL	2.1 ± 1.0	2.2 ± 0.9			
Total/HDL (au)	AG1	3.3 ± 1.1	3.2 ± 1.0	0.434	0.666	0.489
	PL	3.5 ± 1.2	3.5 ± 1.1			
SBP (mmHg)	AG1	115.1 ± 13.6	117.3 ± 13.0	0.288	0.869	0.973
	PL	114.4 ± 11.8	116.5 ± 14.4			
DBP (mmHg)	AG1	73.2 ± 11.1	73.7 ± 9.9	0.459	0.812	0.669
	PL	73.3 ± 6.9	75.0 ± 6.9			
HR (bpm)	AG1	65.5 ± 8.8	68.7 ± 11.6	0.032*	0.961	0.981
	PL	65.4 ± 10.1	68.5 ± 9.2			
Body weight (kg)	AG1	74.5 ± 11.9	74.6 ± 11.8	0.313	0.615	0.684
	PL	76.9 ± 14.6	77.2 ± 15.0			

*Indicates a statistically significant effect/interaction between treatments (*p* ≤ 0.05).

HDL: High-density lipoprotein; VLDL: Very low-density lipoprotein; LDL: Low-density lipoprotein; SBP: Systolic blood pressure; DBP: Diastolic blood pressure; HR: Heart rate.

Only two participants [one in each group (i.e. 6.7% of participants in each group)] reported AEs during this clinical trial. One participant within the PL group reported abdominal pain and diarrhea that were considered moderate intensity and possibly related to the intervention consumed. Another participant within the AG1 group reported bloating that was considered mild in intensity and possibly related to the intervention consumed.

## 4. Discussion

The purpose of this study was to assess the effect of 28 days of AG1^Ⓡ^ supplementation on microbiome structure, GI tolerability, and safety. A main finding was that four weeks of daily AG1^Ⓡ^ supplementation enriched two probiotic taxa, *L. acidophilus* and *B. bifidum*, indicating the probiotics in AG1^Ⓡ^ were able to survive GI passage and reach the large intestine. Additionally, two other taxa (*L. lactis* CH_LC01 and *Acetatifactor* sp900066565 ASM1486575v1 were enriched while *Clostridium* sp000435835 was reduced. Moreover, when analyzing the community function, AG1 showed an enrichment of two functional pathways while diminishing none. Alternatively, the PL enriched six, but diminished five functional pathways. Overall, there was no significant impact on digestive quality of life via DQLQ, VAS, and Bristol in either treatment group over the 4-week supplementation period. However, there may have been a greater improvement in DQLQ score (+62.5%, *p* = 0.058) after four weeks of AG1^Ⓡ^ supplementation as compared to a reduction (−50%) in PL. Furthermore, within the confines of this study design, AG1^Ⓡ^ appeared to be safe for daily consumption as indicated by the absence of significant alterations in clinical safety markers following supplementation.

While there were positive changes in enrichment, α-diversity did not change significantly in the two groups, which is consistent with previous findings that administered probiotics to healthy individuals [2,48–50]. Analysis of the β-diversity indicated clear clustering centered around both the treatment group and the time point. Despite clear clustering based on time, there was no evidence to suggest that heterogeneity shifted due to either treatment which is consistent with previous human [51] and murine [52] studies.

Hence, we used the LEfSe analysis to identify the differences in taxa before and after the administration of each treatment given that LEfSe is the only way to provide a direct assessment of the treatment effect on microbiome structure. Within AG1, four taxa were enriched (e.g. *L. acidophilus, B. bifidum, Acetatifactor* sp900066565 ASM1486575v1, *and L. lactis* CH_LC01) while one taxon (i.e. *Clostridium* sp000435835) was diminished. Enrichment of *L. acidophilus* and *B. bifidum* were not a surprise given they are the same species as the probiotics in AG1^Ⓡ^. These data add to the clinical significance of *L. acidophilus* and *B. Bifidum* probiotic strains, as they have demonstrated the ability to positively shift the microbiome, increase SCFA production (e.g. butyrate and acetate), improve IBS symptoms and stool consistency, and enhance overall quality of life by reducing pain, bloating, urgency, and digestive discomfort in human clinical trials [48,53–55].

Interestingly, a more liberal analysis showed the enrichment of additional health promoting taxa within the AG1 group. These taxa are linked to increased SCFA production and to the ability to cleave glycosidic residues from complex carbohydrate species, resulting in beneficial metabolites through carbohydrate fermentation. These taxa may exert their immune-modulatory effect through the interaction with toll-like receptor 2 which plays a role in maintaining intestinal epithelia barrier integrity [12]. This may have subsequently resulted in an improvement in GI permeability and enhancement of the GI barrier through the upregulation of tight junction proteins or protein kinase signaling pathways that lead the formation of tight junctions [12].

Alternatively, six taxa were enriched while five taxa were diminished in the PL group which may explain maltodextrin’s impact on the gut microbiome [56]. Due to the variability in the PL group, AG1^Ⓡ^ may be better able to stabilize the gut microbiome than maltodextrin. Given the gut microbiome’s association with various physiological functions in the human body, it is crucial to maintain its overall functionality and stability. Maintaining the stability of the microbiome is essential, as it is constantly subjected to stressors such as poor diet, lack of exercise, stress, and environmental toxins [57]. These stressors can lead to a dysbiotic state in the microbiome which is linked to a variety of chronic health conditions such as obesity, diabetes, and cardiovascular disease [58].

However, it is worthwhile to note that after correcting for existing differences at baseline, a total of 19 taxa were enriched and 21 taxa diminished within the AG1^Ⓡ^ treated group. Of note, within *Faecalibacterium prausnitzii*, there appeared to be a strain-specific effect elicited by AG1^Ⓡ^ where four specific strains were either enriched or diminished over the experimental period. *F. prausnitzii* is particularly noteworthy for its ability to produce butyrate, a SCFA closely linked to GI health. Furthermore, studies have shown that the abundance of *F. prausnitzii* can serve as an indicator or biomarker of intestinal health as depletion of *F. prausnitzii* has been associated with multiple health complications and syndromes (e.g. diabetes, IBS, IBD, metabolic syndrome), although the exact relationship as a cause or consequence is not yet fully understood [59]. Additionally, there may have been an increase in key metabolic pathways due to the prebiotics and phytonutrients within AG1^Ⓡ^ that can act as key metabolic substrates used by the microbiome. In return, these may result in increased metabolite production in pathways like the tryptophan metabolism which generate molecules like serotonin that promote normal intestinal regularity. This specific metabolic effect was demonstrated in one of the preclinical studies on AG1^Ⓡ^ [18]. This discovery makes it exciting to postulate that AG1^Ⓡ^ might have a nuanced effect on the gut microbiome that warrants further exploration in the future.

Participants did not report any negative changes in digestive health via VAS questions on stool consistency and bowel frequency which is not surprising given that these were healthy participants, reporting normal stool consistency and bowel frequency at baseline. Likewise, participants did not report any statistically significant changes in digestive symptoms interfering with their quality of life (DQLQ). However, there was a possible difference between the DQLQ change score among groups (*p* = 0.058) where AG1^Ⓡ^improved scores by 62.5% and the PL reduced scores by 50%, on average. Participants in both treatments also reported healthy stools on the Bristol stool chart before and after the intervention, thus there were no statistically significant differences. These data add to the body of clinical support for *L. Acidophilus* species probiotics including a study that enrolled 340 adults with IBS. Previously, *L. Acidophilus* NCFM administered at 1 or 10 billion CFU significantly reduced moderate to severe abdominal pain, supporting its role in mitigating visceral pain through increased analgesic receptor expression [54]. These findings are contradictory to other studies investigating probiotics with more species/strains at a higher dose [60] or with a single strain (i.e. *B. coagulans* or *B. bifidum*) and lower dose (1–2 billion CFU) [48,53,61] as compared to the current investigational product, however the current study examined healthy men and women whereas other trials were conducted on individuals with mild to moderate GI complications. Thus, a follow-up clinical trial conducted on individuals with GI complications may prove beneficial. Nevertheless, the main finding from these data is that AG1^Ⓡ^ is well tolerated and produced no significant GI distress between conditions.

AG1^Ⓡ^ supplementation proved to have a minimal impact on biomarkers of clinical safety via CBC, CMP, lipid panel, and vital signs after four weeks of consumption. The only group by time interactions of note were for a significant decrease in eGFR and a significant increase in creatinine for the PL group. This adds to an established stance on the safety and recommended intake of probiotics and prebiotics [5,9,62]. Lastly, AG1^Ⓡ^ resulted in only one mild adverse event similar in prevalence to the placebo intervention.

Additionally, there were limitations in this study which should be considered in the interpretation of these data. The microbiome data in this study could be enhanced in the future by studying the transcriptome and metabolome to see if alterations in metabolic pathways occurred. Previous reports suggest that maltodextrin is not entirely inert and exhibits an effect on the gut microbiome, however, to date it remains the most commonly utilized placebo in previous studies [48,50,53,61]. Nevertheless, it has questionable validity as a placebo control within clinical trials, and future studies may want to use an alternative to maltodextrin when assessing functional and structural changes in the gut microbiome [56].

In conclusion, AG1^Ⓡ^ supplementation was shown to enrich beneficial taxa in the microbiome, can be consumed safely by healthy adults over four weeks, and has a potential to improve digestive quality of life. While not the aim of this study, further investigation of AG1^Ⓡ^ in a population with GI issues could potentially have a more significant impact on digestive quality of life based on these findings. Overall, our data suggest that the probiotics in AG1^Ⓡ^ survive GI passage to reach the distal GI tract and does not produce any significant negative impact on subjective or objective indices of health.

## Supplementary Material

Supplemental Material
