# Supplementary material for: The effects of AG1® supplementation on the gut microbiome of healthy adults: a randomized, double-blind, placebo-controlled clinical trial
Source: J Int Soc Sports Nutr. 2024 Oct 1;21(1):2409682. doi: 10.1080/15502783.2024.2409682 (PMC11445888; doi:10.1080/15502783.2024.2409682)
Supplement: Supplemental Material [file RSSN_A_2409682_SM9266.zip › Supp/Supplemental Figure B.docx]

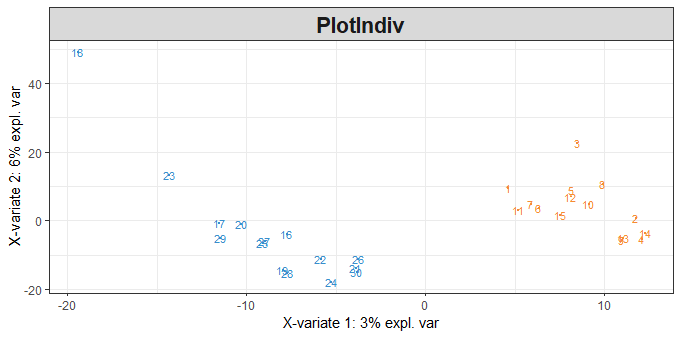


Figure B. A PLS-DA ordination visualizes the effects of treatment and time influenced on com-munity structural heterogeneity for the placebo (maltodextrin) group. Numbers 1 through 15 (orange) refer to the baseline sampling. Numbers 16 through 30 (blue) refer to the sampling point following the experimental phase.
