# Supplementary material for: The effects of AG1® supplementation on the gut microbiome of healthy adults: a randomized, double-blind, placebo-controlled clinical trial
Source: J Int Soc Sports Nutr. 2024 Oct 1;21(1):2409682. doi: 10.1080/15502783.2024.2409682 (PMC11445888; doi:10.1080/15502783.2024.2409682)
Supplement: Supplemental Material [file RSSN_A_2409682_SM9266.zip › Supp/Supplementary Tables.docx]

Table A. Results from the taxonomic LEfSe analysis for the AG1 treated group.

| **Taxon** | **Enrichment Group** | **LDA-Score** | ***P*-value** |
| --- | --- | --- | --- |
| *Bifidobacterium bifidum* | Post AG1 | 2.932 | 0.004 |
| *Lactococcus lactis* CH_LC01 | Post AG1 | 1.885 | 0.008 |
| *Acetatifactor* sp900066565 ASM1486575v1 | Post AG1 | 1.705 | 0.035 |
| *Lactobacillus acidophilus* | Post AG1 | 1.627 | 0.035 |
| *Clostridium* sp000435835 | Pre AG1 | 1.740 | 0.035 |

Table B. Results from the taxonomic LEfSe analysis for the placebo group.

| **Taxon** | **Enrichment Group** | **LDA-Score** | **P-value** |
| --- | --- | --- | --- |
| *Roseburia hominis* MCC695 | Post Placebo | 1.950 | 0.035 |
| *Massilimaliae* | Post Placebo | 1.785 | 0.041 |
| *Massilimaliae timonensis* | Post Placebo | 1.755 | 0.041 |
| *Blautia obeum* MSK 20_67 | Post Placebo | 1.688 | 0.027 |
| *Eubacterium ventriosum* ATCC_27560 | Post Placebo | 1.583 | 0.017 |
| GCA_900066755 sp900066755 | Post Placebo | 1.429 | 0.035 |
| *Clostridiales* | Pre Placebo | 2.472 | 0.027 |
| *Clostridium* | Pre Placebo | 2.470 | 0.027 |
| *Clostridiaceae* | Pre Placebo | 2.468 | 0.027 |
| *Blautia obeum* MSK 20_66 | Pre Placebo | 1.944 | 0.035 |
| *Eubacterium ventriosum* D43t1_170807_H10 | Pre Placebo | 1.621 | 0.035 |

Table C. Results from the taxonomic LEfSe analysis for the AG1 treated group.

| **Taxon** | **Enrichment Group** | **LDA-Score** | **P-value** |
| --- | --- | --- | --- |
| Palmitate biosynthesis - type I fatty acid synthase (PWY_5994) | Post AG1 | 2.252 | 0.024 |
| Guanosine ribonucleotides de novo biosynthesis (PWY_7221) | Post AG1 | 2.260 | 0.029 |

Table D. Results from the taxonomic LEfSe analysis for the placebo (maltodextrin) group.

| **Taxon** | **Enrichment Group** | **LDA-Score** | **P-value** |
| --- | --- | --- | --- |
| Superpathway of menaquinol 8 biosynthesis I (PWY_5838) | Post Placebo | 2.252 | 0.033 |
| Superpathway of menaquinol 12 biosynthesis I (PWY_5898) | Post Placebo | 2.234 | 0.029 |
| Superpathway of menaquinol 11 biosynthesis I (PWY_5897) | Post Placebo | 2.228 | 0.029 |
| Superpathway of menaquinol 13 biosynthesis I (PWY_5899) | Post Placebo | 2.227 | 0.029 |
| Superpathway of demethylmenaquinol 8 biosynthesis I (PWY_5861) | Post Placebo | 2.122 | 0.033 |
| 2-carboxy-1,4-naphthoquinol biosynthesis (PWY_5837) | Post Placebo | 1.840 | 0.029 |
| L-arginine biosynthesis III via N-acetyl-L-citrulline (PWY_5154) | Pre Placebo | 2.529 | 0.044 |
| Inosine-5-phosphate degradation | Pre Placebo | 2.378 | 0.029 |
| Superpathway of pyrimidine deoxyribonucleotides de novo biosynthesis (PWY_7211) | Pre Placebo | 2.226 | 0.042 |
| Pyrimidine deoxyribonucleotides de novo biosynthesis_I (PWY_7184) | Pre Placebo | 2.034 | 0.042 |
| Pyrimidine deoxyribonucleotides de novo biosynthesis_III (PWY_6545) | Pre Placebo | 2.033 | 0.042 |
