# Supplementary figures and images for: The effects of AG1® supplementation on the gut microbiome of healthy adults: a randomized, double-blind, placebo-controlled clinical trial
Source: J Int Soc Sports Nutr. 2024 Oct 1;21(1):2409682. doi: 10.1080/15502783.2024.2409682 (PMC11445888; doi:10.1080/15502783.2024.2409682)

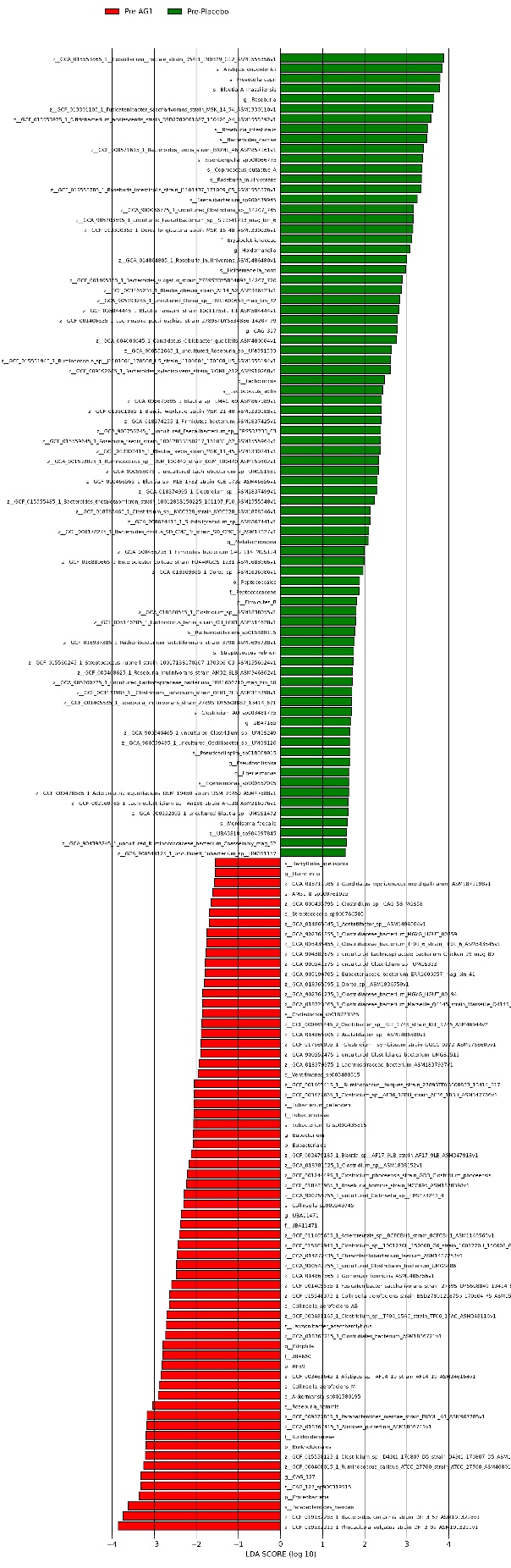


Figure C. Pre (before the intervention) taxa differences for AG1 and PL

Supplement: Supplemental Material [file RSSN_A_2409682_SM9266.zip › Supp/Supplemental Figure C.docx]

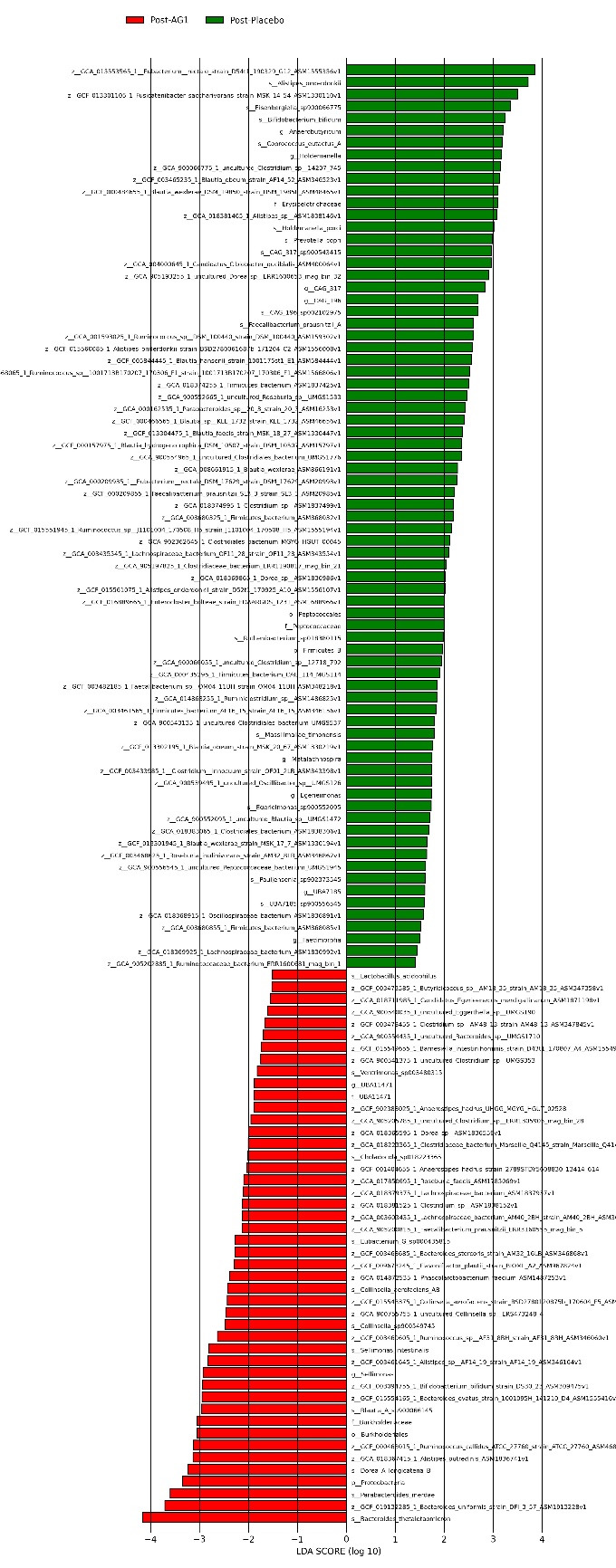


Figure D. Post (after the intervention) taxa differences for AG1 and PL

Supplement: Supplemental Material [file RSSN_A_2409682_SM9266.zip › Supp/Supplemental Figure D.docx]
